# JunB Is Critical for Survival of T Helper Cells

**DOI:** 10.3389/fimmu.2022.901030

**Published:** 2022-06-28

**Authors:** Tsunghan Hsieh, Daiki Sasaki, Naoyuki Taira, Hsiaochiao Chien, Shukla Sarkar, Yu Seto, Mio Miyagi, Hiroki Ishikawa

**Affiliations:** Immune Signal Unit, Okinawa Institute of Science and Technology Graduate University, Okinawa, Japan

**Keywords:** clonal expansion, apoptosis, JunB, T helper cell, AP-1 – activator protein 1

## Abstract

Clonal expansion and differentiation of various T helper subsets, such as Th1, Th2, and Th17 cells, depend on a complex of transcription factors, IRF4 and a BATF-containing AP-1 heterodimer. A major BATF heterodimeric partner, JunB, regulates Th17 differentiation, but the role of JunB in other T helper subsets is not well understood. Here we demonstrate that JunB is required for clonal expansion of Th1, Th2 and Th17 cells. In mice immunized with lipopolysaccharide (LPS), papain, or complete Freund’s adjuvant (CFA), which induce predominantly Th1, Th2 and Th17 cells, respectively, accumulation of antigen-primed, *Junb*-deficient CD4^+^ T cells is significantly impaired. TCR-stimulated *Junb*-deficient CD4^+^ T cells are more sensitive to apoptosis, although they showed largely normal proliferation and cellular metabolism. JunB directly inhibits expression of genes involved in apoptosis, including *Bcl2l11* (encoding Bim), by promoting IRF4 DNA binding at the gene locus. Taken together, JunB serves a critical function in clonal expansion of diverse T helper cells by inhibiting their apoptosis.

## Introduction

Differentiation of specific CD4^+^ T helper cells is a key event for adaptive immune responses. Upon recognition of antigens, naïve CD4^+^ T cells differentiate into T helper subsets, such as Th1, Th2, and Th17 cells, which control different types of adaptive immunity. Depending on the cytokine signals that antigen-primed CD4^+^ T cells receive, specific STATs (signal transducers and activator transcription) are activated (1) and induce lineage-specifying transcription factors (T-bet in Th1, GATA3 in Th2, and RORγt in Th17), which are critical for expression of signature cytokines, such as IFN-γ in Th1, IL-4 and IL-13 in Th2, and IL-17 in Th17 subsets (2).

A trimeric transcription factor complex composed of IRF4 and a BATF-containing AP-1 dimer is pivotal in clonal expansion and differentiation of T helper cells, including Th1, Th2, Th9, Th17, and T follicular helper (Tfh) cells (3–12). IRF4 and BATF not only control expression of lineage-specific genes, such as lineage-specifying transcription factors and effector cytokines, but also regulate the transcriptional program that is required for effector T cell survival, proliferation, and metabolic reprogramming. Binding of IRF4 and BATF to DNA loci containing AICE (AP-1-IRF composite element) motifs promotes chromatin accessibility (13), thereby inducing expression of target genes (14, 15). Expression levels of BATF and IRF4 are correlated with the strength of TCR signaling and affect the target specificity of IRF4 and BATF (16).

JunB is a major heterodimeric partner for BATF in CD4^+^ helper T cell differentiation (14, 15, 17). We and others have reported that JunB is required for generation of pathogenic Th17 cells that cause autoimmunity, but not for gut-resident homeostatic Th17 cells (18–20). JunB also controls effector Treg homeostasis and immune suppressive functions (14, 15, 21–23). Furthermore, JunB promotes expression of cytokines specific to Th2 and Th9 cells (17, 24). Thus, JunB likely contributes to differentiation of various CD4^+^ T helper subsets. However, *in vivo* roles for JunB in diverse T helper subsets are not well understood.

In this study, we investigated the role of JunB in CD4^+^ T helper differentiation in mice immunized with LPS, papain, or complete Freund’s adjuvant (CFA) and found that accumulation of antigen-primed *Junb*-deficient CD4^+^ T cells was dramatically impaired in all immunization protocols. Loss of JunB compromised survival of TCR-stimulated CD4^+^ T cells under Th1-, Th2-, and Th17-polarizing conditions. RNA-seq and chromatin immunoprecipitation PCR (ChIP-PCR) analyses revealed that JunB promoted IRF4 binding to the *Bcl2l11* gene locus, thereby inhibiting Bim expression. Taken together, our study uncovers a critical function of JunB in clonal expansion of various T helper subsets.

## Materials and Methods

### Mice

Floxed *Junb* (*Junb^fl/fl^*) mice have been described previously (19). *Cd4^Cre^* (stock# 017336), OT-II (stock# 004194) and B6SJL (stock# 002014) mice were obtained from the Jackson Laboratory (Bar Harbor, ME, USA). All mice were of a C57BL/6 background and were maintained under specific pathogen-free conditions. Sex-matched, 6–12-week-old mice were used for experiments. All animal experimental protocols were approved by the Animal Care and Use Committee at Okinawa Institute of Science and Technology Graduate University.

### Isolation of Naïve CD4^+^ T Cells

Murine naïve CD4^+^ T cells were purified from pooled spleens by negative selection using mouse naïve CD4^+^ T cell selection kits (480039; Biolegend, San Diego, CA, USA), in accordance with manufacturer instructions. Flow cytometry analysis confirmed that the purity of CD4^+^CD25^-^CD62L^hi^CD44^lo^ cells ranged from 90% to 95%.

### Adoptive Transfer

Naïve CD4^+^ T cells isolated from *Junb^fl/fl^* OT-II or *Junb ^fl/fl^Cd4^Cre^* OT-II mice (CD45.2^+^) were mixed with naïve CD4^+^ T cells isolated from congenic OT-II mice (CD45.1^+^CD45.2^+^) at a ratio of 2:1. Cells were labelled with 2 μL CFSE (423801; Biolegend) in 1 mL of phosphate-buffered saline (PBS) for 20 min at room temperature and washed with PBS containing 2% fetal bovine serum (FBS, FB-I061; Biosera, Manila, Philippines). CFSE-labeled cells (3 x 10^6^ cells per mouse) were intravenously injected into congenic recipient B6SJL mice (CD45.1^+^).

### Immunization

One day after adoptive transfer, mice were anesthetized with isoflurane and immunized with 20 μg of OVA peptide 323-339 (ISQAVHAAHAEINEAGR, GL Biochem, Shanghai, China) emulsified in 100 μL complete Freund’s adjuvant (CFA) or mixed with 10 μg of lipopolysaccharides (LPS) from *Escherichia coli* O111 (L4391; Sigma, St. Louis, MO, USA) or 40 μg papain (P5306; Sigma). CFA was prepared from 100 μL incomplete Freund’s adjuvant (263910; BD, Franklin Lakes, NJ, USA) and 1 μg desiccated *Mycobacterium tuberculosis* H37 Ra (231141; BD) according to the manufacturer’s instructions and emulsified with OVA peptide using an ultrasonic homogenizer (VP-050; TAITEC, Koshigaya, Saitama, Japan) on ice for 30-45 min. LPS and papain were dissolved in PBS and mixed with OVA peptide at room temperature before immunization. For immunization with CFA or LPS, mice were injected subcutaneously on each side close to the base of tail. For immunization with papain, mice were injected intranasally with 40 μg papain on two consecutive days.

### Cell Culture

Purified naïve CD4^+^ T cells were cultured in 24-well (4 x 10^5^ cells per well), 48-well (2 x 10^5^ cells per well) or 96-well (1 x 10^5^ cells per well) plates coated with 5 μg/mL anti-CD3ϵ antibody (145-2C11; Biolegend) in IMDM medium (12440-061; Invitrogen) containing 1 μg/mL anti-CD28 antibody (37.51; Biolegend), 10% FBS, 1x streptomycin-penicillin (containing 100 U/mL penicillin and 100 μg/mL streptomycin, P4333; Sigma), and 55 μM β-mercaptoethanol (20985-023; Invitrogen, Waltham, MA, USA). In addition, the following cytokines and antibodies were added in each polarizing condition: 20 ng/mL IL-2 (570402; Biolegend), 1 μg/mL anti-IFN-γ (XMG1.2; Biolegend), and 1 μg/mL anti-IL-4 (11B11; Biolegend) for Th0; 20 ng/mL IL-2, 20 ng/mL IL-12 (577002; Biolegend), and 1 μg/mL anti-IL-4 for Th1; 20 ng/mL IL-2, 100 ng/mL IL-4 (574306; Biolegend), and 1 μg/mL anti-IFN-γ for Th2; 20 ng/mL IL-6 (575706; Biolegend), and 3 ng/mL TGF-β1 (100-21C; PeproTech, Cranbury, NJ, USA) for Th17. Polarized cells were harvested for further analysis at the indicated time points.

### Antibodies

For flow cytometry analysis, the following antibodies were used at a 1:100 dilution: anti-CD3 (17A2; Biolegend), anti-CD4 (GK1.5; Biolegend), anti-CD25 (PC61; Biolegend), anti-CD44 (IM7; Biolegend), anti-CD62L (MEL-14; Biolegend), anti-CD45.1 (A20; Biolegend), anti-CD45.2 (104; Biolegend), anti-FasL (MFL3; Biolegend), anti-IL-17A (TC11-18H10.1; Biolegend), anti-IFN-γ (XMG1.2; Biolegend), anti-JunB (C-11; Santa Cruz Biotechnology, Santa Cruz, CA, USA), anti-GATA3 (16E10A23; Biolegend), anti-ROR-γt (Q31-378; BD), anti-T-bet (4B10; Biolegend), and anti-rabbit IgG (Poly4064; Biolegend). For ChIP analyses, anti-JunB (1 μg per ChIP, 210; Santa Cruz), anti-BATF (1 μg per ChIP, WW8; Santa Cruz), anti-IRF4 (1 μg per ChIP, M-17; Santa Cruz) and rabbit IgG (1 μg per ChIP, I5006; Sigma) were used.

### Flow Cytometry

**Figure 1:**
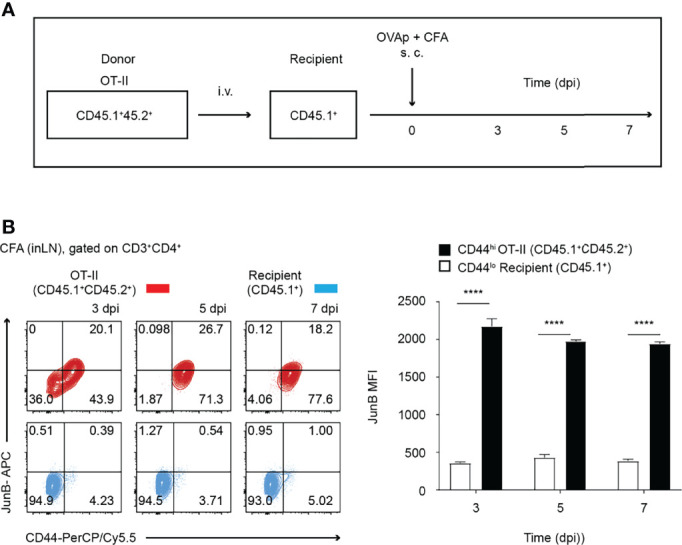
JunB is expressed in antigen-primed CD4^+^ T cells *in vivo*. 2 x 10^6^ naive OT-II cells (CD45.1^+^CD45.2^+^) were transferred to congenic recipient mice (CD45.1^+^), followed by immunization with OVA_323-339_ peptides emulsified in CFA. At the indicated days post-immunization (dpi), cells were harvested from inaugural lymph nodes and analyzed. **(A)** Immunization scheme. *i.v.* intravenous injection, *s.c.* subcutaneous injection. **(B)** Flow cytometry analysis of JunB expression in transferred OT-II cells (CD3^+^CD4^+^CD45.1^+^CD45.2^+^, shown in red) and recipient naïve CD4^+^ T cells (CD3^+^CD4^+^CD45.1^+^, shown in blue) at the indicated dpi. Bar graphs show median fluorescence intensity (MFI) of JunB expression levels in CD44^hi^ OT-II cells and CD44^lo^ recipient cells. Error bars indicate s.d. (n =3). ****p < 0.0001, (unpaired two-tailed Student’s t-test).

**Figure 2:**
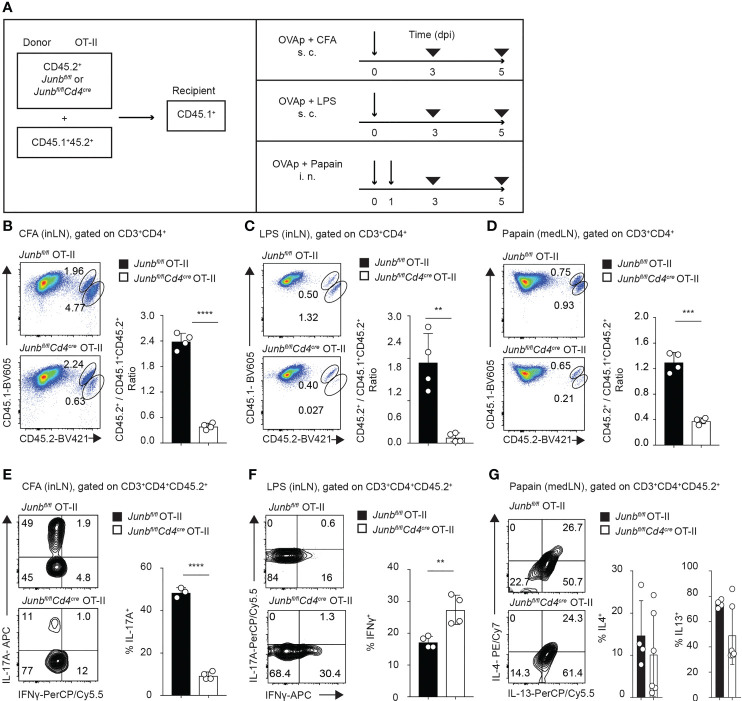
JunB promotes accumulation of antigen-primed CD4^+^ T cells *in vivo*. Naive *Junb^fl/fl^* OT-II or *Junb^fl/fl^Cd4^Cre^* OT-II cells (CD45.2^+^) were co-transferred with congenic wild-type OT-II cells (CD45.1^+^CD45.2^+^) at a 2:1 ratio into congenic recipient mice (CD45.1^+^). One day later, mice were immunized with OVA_323-339_ peptides emulsified in CFA or mixed with LPS or papain. At 5 dpi, cells were isolated from draining lymph nodes and analyzed by flow cytometry. **(A)** Immunization scheme. *i.v.* intravenous injection, *s.c.* subcutaneous injection, *i.n.* intranasal injection. **(B–D)** Flow cytometry analysis of CD45.1 and CD45.2 expression in CD3^+^CD4^+^ T cells isolated from mice immunized with CFA **(B)**, LPS **(C)**, and papain **(D)**. Bar graphs show the ratio of *Junb^fl/fl^* OT-II or *Junb^fl/fl^Cd4^Cre^* OT-II cells (CD45.2^+^) vs co-transferred OT-II cells (CD45.1^+^CD45.2^+^). **(E–G)** Flow cytometry analysis of IL-17A and IFN-γ expression **(E, F)** and IL-4 and IL-13 expression **(G)** in OT-II cells (CD3^+^CD4^+^CD45.2^+^) isolated from mice immunized with CFA **(E)**, LPS **(F)**, and papain **(G)**. Bar graphs indicate the percentage of cells expressing the indicated cytokines in *Junb^fl/fl^* OT-II or *Junb^fl/fl^Cd4^Cre^* OT-II cells. **(B–F)** Error bars indicate s.d. (n = 4-6 mice per group). **p < 0.01, ***p < 0.001, ****p < 0.0001, (unpaired two-tailed Student’s t-test). Data represent two independent experiments.

**Figure 3:**
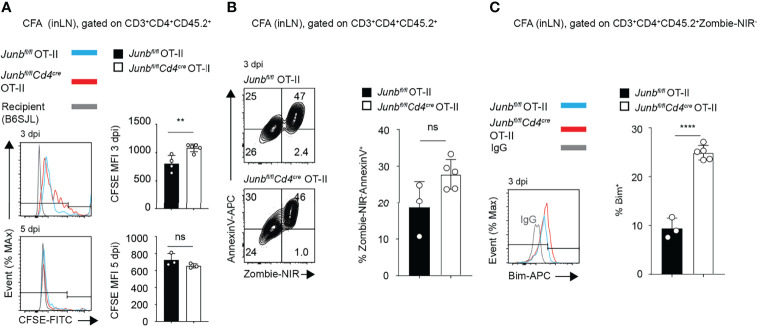
JunB is required for survival of CD4+ effector T cells *in vivo*. CFSE-labelled *Junbfl/fl* OT-II or *Junbfl/flCd4cre* OT-II cells (CD45.2+) were intravenously transferred to congenic recipient mice (CD45.1+), followed by immunization with OVA323-339 peptides emulsified in complete Freund’s adjuvant (CFA). At indicated days post-immunization (dpi), cells were harvested from inguinal lymph nodes and analyzed. Flow cytometry analysis of CFSE dilution **(A)**, percentages of Zombie-NIR^-^AnnexinV^+^ cells **(B)**, and expression of Bim **(C)**, as measured by median fluorescence intensity (MFI) **(A–C)** Error bars indicate s.d. (n = 4-6 wells per group). ns: non-significant, (*p* > 0.05) **p < 0.01, ****p < 0.0001, (unpaired two-tailed Student’s t-test). Data represent two independent experiments.

For analysis of cell surface molecules, cells were stained with antibodies and Zombie-NIR (1:400, 423106; Biolegend) in PBS containing 2% FBS for 30 min on ice. For analysis of intracellular molecules, cells were stained with a Foxp3 Staining Buffer Set (00-5253-00; eBioscience) according to the manufacturer’s protocol. For analysis of intracellular cytokines, cells were re-stimulated with 100 ng/mL phorbol 12-myristate 13-acetate (P8139; Sigma) and 1 μg/mL ionomycin (I0634; Sigma) in the presence of 10 μg/mL brefeldin A (420601; Biolegend) for 4 h, and then stained with a Foxp3 Staining Buffer Set. For analysis of cells isolated from lymph nodes (**Figures 1**–**3**), cells were incubated with anti-CD16/CD32 (1:100, 93; Biolegend) before antibody staining. Gating strategies for flow cytometry analysis are described in **Supplementary Figure 7**.

### Preparation of Cas9 Ribonucleoprotein Complex

Cas9 ribonucleoprotein (RNP) complex was prepared as described in detail previously (25). In short, 1 nmol of Alt-R crRNA targeting *Junb* (crJunB, CGCCCGGATGTGCACGAAAA, Integrated DNA Technology, Singapore) or negative control Alt-R crRNA (crNTC, 1072544; Integrated DNA Technology) was first mixed with 1 nmol of Alt-R tracrRNA (1072535; Integrated DNA Technology) in 20 μL at room temperature for 10 min. RNA mixture was then annealed by heating at 95°C for 5 min in a thermocycler (TP600; Takara, Tokyo, Japan) and slowly cooled to 25°C. For one nucleofection reaction, RNP complex was prepared by mixing 150 pmol crRNA:tracRNA duplex with 60 pmol Cas9 protein (A36498; Invitrogen) at room temperature for 10 min right before nucleofection.

### Nucleofection of Naïve CD4^+^ T Cells

Nucleofection of naïve CD4^+^ T cells was performed using a P4 primary cell nucleofector kit (V4XP-4024; Lonza) following the manufacturer’s protocol (25). Up to 1x10^7^ purified naïve CD4^+^ T cells from C57BL/6 mice were washed with PBS, suspended in 20 μL P4 primary cell nucleofector solution and mixed with 5 μL of RNP complex at room temperature for 2 min in a round-bottom 96-well plate. The cell/RNP mix was transferred to nucleofection cuvette strips, and CD4^+^ T cells were electroporated using Lonza 4D Nucleofector X unit (program code: DS137). After nucleofection, 200 μL prewarmed IMDM medium was added to a cuvette to transfer cells into flat-bottom 96-well plates. 1 x 10^6^ cells were then rested in IMDM medium containing 5 ng/mL IL-7 (577802; Biolegend), 10% FBS, 1 x streptomycin-penicillin, and 55 μM β-mercaptoethanol for 72 h before polarization.

### Seahorse Assay

Oxygen consumption rate (OCR) and extracellular acidification rate (ECAR) were assayed with mito stress (103015-100; Agilent Technologies, Santa Clara, CA, USA) and glycolysis stress kits (103020-100; Agilent Technologies), respectively. Naïve CD4^+^ T cells were cultured under Th1-, Th2- and Th17-polarizing conditions for 48 h. Polarized cells were washed with PBS, transferred to an analysis plate (2 x 10^5^ cells per well) coated with 50 μL of 2% gelatin (G1890; Sigma), and pre-incubated at 37 °C for 1 h. To measure OCR, cells were incubated with XF base medium supplemented with 1 mM pyruvate (11360070; Gibco), 2 mM glutamine (A2916801; Gibco) and 10 mM glucose (A2494001; Gibco) and were treated with 1.5 μM oligomycin, 1 μM fluorocarbonyl cyanide phenylhydrazone (FCCP) and 0.5 μM rotenone/antimycin A mix (103015-100; Agilent Technologies). To measure ECAR, cells were incubated with XF base medium supplemented with 2 mM glutamine and were treated with 10 mM glucose, 1 μM oligomycin, and 50 mM 2-deoxyglucose (2-DG) (103020-100; Agilent Technologies). Cells were analyzed using a Seahorse XFe96 analyzer (Seahorse Bioscience, North Billerica, MA, USA), according to the manufacturer’s instructions.

### RNA-Seq

Naïve CD4^+^ T cells were cultured under Th0-, Th1- and Th2-polarizing conditions for 48 h. Then, cells were stained with Zombie-NIR (1:400, 423105; Biolegend), and viable cells were sorted with FACS. RNA samples were prepared using Trizol (Invitrogen) with a Qiagen RNAeasy kit (Qiagen, Hilden, Germany). Total RNA was provided to the OIST DNA sequencing section for library preparation and sequencing. cDNA libraries for RNA-Seq were prepared with a NEBNext Ultra II Directional RNA Library Prep Kit for Illumina (E7760L; New England BioLabs, Ipswich, MA, USA) and purified using Agencourt AMPure XP beads (A63880; Beckman Coulter) following the manufacturer’s instructions. Adapter dimers in cDNA libraries were removed with a LabChip NGS 3K reagent kit (CLS960013; PerkinElmer, Waltham, MA, USA) and confirmed using a TapeStation (Agilent). Purified cDNA libraries were quantified with droplet digital PCR (BioRad QX-200 system). Sequencing was performed on an Illumina NovaSeq 6000 to generate 150-nucleotide, paired-end reads at a read depth of ≥20 million reads per sample. Each experiment contained 3 or 4 biological replicates. Male and female mice were used in equal numbers in library preparation.

### Differential Gene Expression Analysis

Raw reads from RNA-Seq were first trimmed with Cutadapt 2.10 (26). Trimmed reads were then directly mapped to the UCSC mouse genome *mm10*, and transcripts were quantified with Salmon 1.3.0, using default settings. To provide gene annotation, a mouse genome index was used during transcript quantification with a k value of 31 (27). After transcript quantification, counts of each transcript were first normalized within and between samples to obtain TPM (Transcripts Per kilobase Million). Differential gene expression analysis was conducted with DeSeq2 (28). Three or four independent biological samples were used for the analysis. Genes that were differentially expressed in *Junb*-deficient vs control cells (log2 Fold change < -0.5 or > 0.5, p value < 0.05, base mean > 100) were selected for biological process analysis using DAVID GO (29).

### ChIP-Seq and ChIP-PCR

ChIP-Seq samples were prepared using a SimpleChIP Plus Enzymatic Chromatin IP Kit (9005S; Cell Signaling Technology, Danvers, MA, USA) as previously described (19). Naïve CD4^+^ T cells from *Junb^fl/fl^* mice were cultured under Th1-polarizing conditions. After 48 h, activated cells (1-2 x 10^6^ per ChIP-seq) were cross-linked in culture medium containing 1% formaldehyde at room temperature for 10 min, and glycine solution was added to stop the reaction. Then fixed cells were lysed, and nuclei were isolated and treated with micrococcal nuclease (0.00313 μL/mL) for 20 min at 37°C. The nuclease reaction was stopped by adding 0.05 M ethylenediaminetetraacetic acid (EDTA). Samples were then sonicated to disrupt nuclear membranes and centrifuged to collect supernatants containing chromatin. Chromatin solutions were incubated with 1 μg of antibodies overnight at 4°C with rotation, and complexes of antibodies and chromatin were collected with Dynabeads Protein G (10004D; Invitrogen). Beads were washed with low-salt and high-salt solutions five times and three times, respectively, and incubated for 5 min for each washing at 4°C. Chromatin was eluted, de-cross-linked following the manufacturer’s instructions, and purified by phenol/chloroform extraction. The resultant DNA was used for generation of a sequencing library for ChIP-seq or for ChIP-PCR analysis.

To generate DNA sequencing libraries for ChIP-seq, DNA was blunt-ended and ligated with adaptors using a KAPA Hyper Prep Kit (KK8500; Sigma Aldrich, St. Louis, MI, USA). Adaptor-ligated DNA was then cleaned with an Agencort AMPure XP (A63880; Beckman Coulter, Wilmington, MA, USA) at a 1.8 x DNA ratio, amplified by PCR, and purified using the AMPure XP at a 1.2 x DNA ratio. Library DNA was size-selected using a 2% agarose gel cassette of Blue Pippin (Sage Science, Beverley, MA, USA) for a target size range 150-300 bp and quantified with droplet digital PCR (QX-200; BioRad, Hercules, CA, USA). Sequencing was performed on an Illumina HiSeq 4000 to generate 150-nucleotide, single-end reads at a read depth of at least 20 million reads per sample. Each ChIP-seq library was prepared from cells collected from 4 mice. Male and female mice were used in equal numbers for library preparation.

For ChIP-PCR, DNA was amplified by quantitative real-time PCR (qPCR) with primers specific for JunB-bound regions of interest at the *Bcl2l11* locus. Primer sequences are as follows: ECR3: CAGCTCACCACCAGTCACAT, GGTGTAGAGAGCAGAAGTCGT; ECR5: TGCCTCTGTGTCAGCACTCT, ACCCAGGATCTCATGTTTGC; and ECR19: AGGCTGCAAGGATACTGTGTTG, TGGAACCAATTGTGTCACACCC. qPCR was performed by using KAPA SYBR FAST Universal mix (KK4602; Sigma Aldrich) and the Ct value was read with StepOne Plus (ABI).

### ChIP-Seq Peak Calling, Annotation, and Visualization

Raw reads of ChIP-Seq obtained as described above were trimmed using Cutadapt 2.10 (26). Trimmed reads were then mapped to the mouse genome *mm10* by calling Bowtie2 2.3.4.3 in TopHat2 2.1.1 (27, 30). Peaks were called for each sample replicate using Homer 4.11 with default parameters (FDR < 0.001). For annotation, peaks were assigned to the nearest genes using the annotatePeaks function in Homer v4.11. To visualize peaks, in Homer v4.11, a mapped read tag directory was first created by calling the makeTagDirectory function and a bed graph file was generated based on this Tag directory (30). Higher than or equal to 50% reciprocal overlapping regions between ChIP-seq peaks for JunB, BATF and IRF4 were identified using bedtools v2.30 (31).

### Motif scan

JunB-binding motifs across the mouse genome (UCSC ver. mm10) were identified using the scanMotifGenomeWide.pl function of Homer v4.11. The AP-1-binding motif, RATGASTCAT, was used for this motif scan. Genomic regions containing AP-1 motifs were assigned to their nearest genes using the annotatePeaks function of Homer v4.11 (30).

### Prediction of Direct JunB Target Genes

To predict direct JunB target genes, using RNA-seq data, we first defined genes that were differentially expressed in *Junb*-deficient vs control cells (log2 Fold change < -0.5 or > 0.5, p value < 0.05, base mean > 100) as JunB target genes. Next, regulatory potential (RP) scores of ChIP-seq peaks and AP-1 motifs locating within ± 100 kb from transcription starting sites (TSS) of JunB target genes were calculated (32): $\text{RP score}=\sum_{i=1}^{k}e^{-\left(0.5+4\Delta i\right)}\;$ , where k equals the number of all binding peaks/motifs within ± 100 kb of the gene (32). Δ is the distance to the TSS of the gene, normalized to 100 kb. For example, Δ = 1 means the ChIP-seq peak or AP-1 motif is within 100 kb from TSS of the gene. A higher RP score indicates a greater density of ChIP-seq peaks or AP-1-binding motifs within ± 100 kb of the TSS of the nearest gene. To verify our prediction, we performed BETA analysis, as described in previous studies (33, 34). Genes with non-zero BETA scores represent a potential direct JunB target.

### Identification of Evolutionarily Conserved Regions at *Bcl2l11* Locus

Evolutionarily conserved regions (ECRs) located ± 50 kb within the mouse *Bcl2l11* TSS region were identified by comparing the *Bcl2l11* DNA sequence between humans (UCSC ver. hg19) and mice (UCSC ver. mm10) genome *via* the evolutionarily conserved region browser (35). Each mouse ECR is 200-bp long and has 75% similarity with the human DNA sequence.

### Statistical Analysis

Unpaired, two-tailed, Student’s t-tests, two-way analysis of variance (two-way ANOVA), and Tukey tests were performed using Prism software (GraphPad). P values < 0.05 were considered statistically significant.

### Data and Code Availability

The RNA-Seq and ChIP-Seq data for this study can be found in the Gene Expression Omnibus with primary accession number GSE172490 [https://www-ncbi-nlm-nih-gov.ezproxy.u-pec.fr/geo/query/acc.cgi?acc=GSE172490]. All command lines and scripts used in this research are available on: https://github.com/oistishikawa/ChIP_Seq and https://github.com/oistishikawa/RNA_Seq.

## Results

### JunB Is Essential for Clonal Expansion of T Helper Cells *In Vivo*

To understand the role of JunB in differentiation of T helper subsets *in vivo*, we first analyzed expression of JunB in CD4^+^ T cells activated upon immunization. We purified naïve (CD62L^hi^ CD44^lo^) ovalbumin (OVA)-specific CD4^+^ T cells (OT-II T cells) from OT-II transgenic mice on a CD45.1^+^ 45.2^+^ background and intravenously transferred those cells into mice on a CD45.1^+^ background (**Figure 1A**). One day later, we immunized mice with OVA_323-339_ peptides emulsified in CFA. At 3, 5, and 7 days post-immunization (dpi), we analyzed expression levels of JunB in CD4^+^ T cells isolated from inaugural lymph nodes and found that JunB expression was significantly increased in activated OT-II T cells (CD44^hi^) and was maintained until 7 dpi (**Figure 1B**).

We next sought to determine effects of JunB deletion on T helper differentiation in mice immunized with various adjuvants. To this end, we co-transferred naïve OT-II T cells isolated from *Junb^fl/fl^* OT-II (control OT-II) or *Junb^fl/fl^Cd4^Cre^* OT-II (*Junb*-deficient OT-II) mice (CD45.2^+^) together with congenic OT-II T cells (CD45.1^+^ 45.2^+^) at a 2:1 ratio into recipient mice (CD45.1^+^). One day later, we immunized mice with OVA_323-339_ peptides together with either CFA, LPS, or papain (**Figure 2A**). In all immunization conditions tested, accumulation of *Junb*-deficient OT-II T cells at 5 dpi was severely impaired (**Figure 2B–D**).

Immunization with CFA, LPS, and papain mainly induced accumulation of OT-II cells expressing cytokines for Th17 (IL-17A), Th1 (IFN-γ), and Th2 cells (IL-4 and IL-13), respectively (**Figurse 2E–G**). Consistent with previous reports (18–20), in mice immunized with CFA, the percentage of IL-17A-expressing cells was significantly lower in *Junb*-deficient OT-II T cells than in controls (**Figure 2E**). In contrast, the percentage of IFNγ-expressing cells in *Junb*-deficient OT-II T cells was increased in mice immunized with LPS (**Figure 2F**). There was no difference in either IL-4 or IL-13 expression between control and *Junb*-deficient OT-II T cells in mice immunized with papain (**Figure 2G**). Taken together, these data suggest that JunB promotes clonal expansion of CD4^+^ T helper cells, regardless of the context of inflammation.

### JunB Is Required for Survival of T Helper Cells

To understand the cause of defective accumulation of *Junb*-deficient OT-II T cells, we analyzed their proliferation and viability in mice immunized with CFA. In a CFSE dilution assay, at 3 dpi, *Junb*-deficient OT-II T cells showed only a slight delay of proliferation, but at 5 dpi there was no difference in proliferation between *Junb*-deficient OT-II T cells and controls (**Figure 3A**). There was no significant difference in the frequency of early apoptotic cells (Zombie-NIR^-^Annexin V^+^) between *Junb*-deficient OT-II cells and controls (**Figure 3B**), but the percentage of cells expressing a pro-apoptotic molecule, Bim, was significantly higher in *Junb*-deficient OT-II T cells (**Figure 3C**).

**Figure 4:**
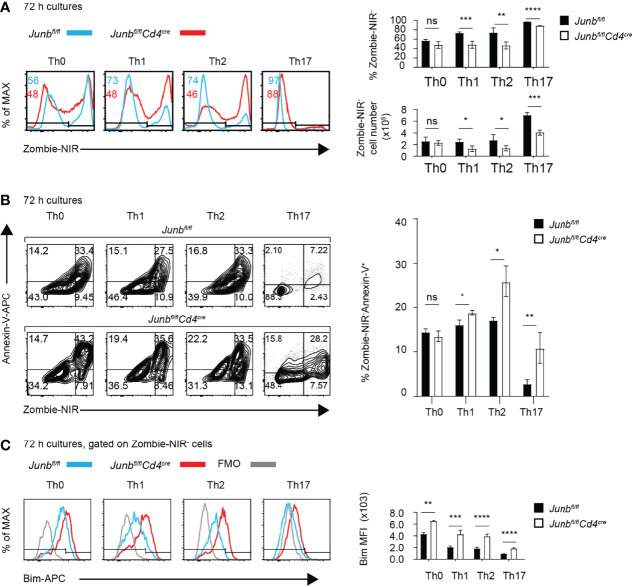
JunB is required for survival of TCR-stimulated CD4^+^ T cells. Naive CD4^+^ T cells isolated from *Junb^fl/fl^* or *Junb^fl/fl^Cd4^Cre^* mice were cultured in differentiation conditions for Th0, Th1, Th2, and Th17 cells and were analyzed by flow cytometry at indicated time points. **(A)** Zombie-NIR staining of cells cultured for 72 h. Numbers in histograms indicate average percentages of Zombie-NIR^-^ cells. Bar graphs show percentages (top right) and absolute numbers (bottom right) of Zombie-NIR^-^ cells. **(B)** Zombie-NIR and Annexin-V staining of cells cultured for 72 h. The bar graph (left) indicates percentages of Zombie-NIR^-^ Annexin-V^+^ cells. **(C)** Analysis of Bim expression in cells cultured for 72 h. The bar graph indicates median fluorescence intensity (MFI) of Bim. **(A–C)** Error bars indicate s.d. (n = 4-6 wells per group). ns, non-significant (*p* > 0.05), *p < 0.05, **p < 0.01, ***p < 0.001, ****p < 0.0001, (unpaired two-tailed Student’s t-test). Data represent two independent experiments.

Unaltered frequency of apoptotic cells in *Junb*-deficient OT-II T cells, despite increased Bim expression, might be due to phagocytic clearance of apoptotic cells *in vivo*. Therefore, we further evaluated effects of JunB defect on survival of differentiating T helper cells *in vitro*. We activated naïve CD4^+^ T cells isolated from *Junb*-deficient or control mice with anti-CD3 and anti-CD28 antibodies in the presence of cytokines that promote differentiation of Th0, Th1, Th2, or Th17 subsets. 72 h after activation, *Junb*-deficient CD4^+^ T cells exhibited a significant decrease in the percentage and absolute number of living cells under Th1-, Th2- and Th17-polarizing conditions, but not Th0-polarizing conditions (**Figure 4A**). In contrast, we did not observe any significant effects of JunB deficiency on proliferation of differentiating T helper cells (**Supplementary Figure 1**). Annexin V staining further demonstrated an increase of living apoptotic cells (Annexin V^+^ Zombie-NIR^-^) at 72 h after activation, but not at either 48 or 96 h, in *Junb*-deficient CD4^+^ T cells under Th1-, Th2-, and Th17-polarizing conditions (**Figure 4B** and **Supplementary Figure 2A**). Consistent with our *in vivo* observation, JunB deficiency upregulated Bim under all Th-polarizing conditions tested (**Figure 4C**).

As apoptosis was affected by the imbalance between Bim and the anti-apoptotic molecule Bcl2 (36), we also examined the expression of Bcl2 in *Junb*-deficient CD4^+^ T cells. JunB deficiency moderately decreased the expression of Bcl2 in cells differentiated under Th1 -polarizing conditions (**Supplementary Figures 2B, C**). JunB deficiency increased the Bim/Bcl2 ratio, reflecting the increase of Bim, in all Th-polarizing conditions tested (**Supplementary Figure 2D**). There was no significant difference in the degrees of increase caused by JunB deficiency in the Bim/Bcl2 ratio among different T helper cells.

To exclude the possibility that the impaired survival of *Junb*-deficient CD4^+^ T cells was due to artificial effects of our Cre/loxP *Junb* knockout model, we evaluated the effect of CRISPR-mediated JunB knockout on survival of T helper cells. We electroporated naïve CD4^+^ T cells isolated from wild-type mice with guide RNAs targeting *Junb* and Cas9 proteins (25) and then activated cells under Th1-, Th2- and Th17-polarizing conditions. Transduction of a guide RNA targeting *Junb* resulted in an increase of cells expressing low levels of JunB (JunB^lo^) (**Supplementary Figure 3A**). Under all differentiation conditions, JunB^lo^ cells exhibited significant decreases of cell survival (**Supplementary Figure 3B**). Together, these data indicate a critical role for JunB in survival of Th1, Th2 and Th17 cells.

### Metabolic Reprogramming in *Junb*-Deficient T Helper Cells

**Figure 5:**
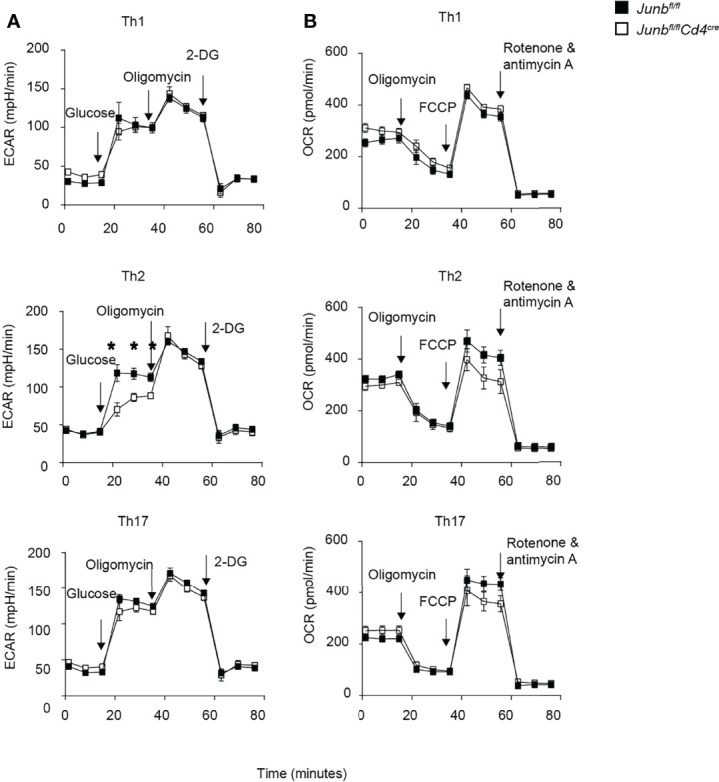
Metabolic reprogramming in *Junb*-deficient CD4^+^ T cells. Naive CD4^+^ T cells isolated from *Junb^fl/fl^* or *Junb^fl/fl^Cd4^Cre^* mice were cultured under Th1-, Th2- and Th17-polarizing conditions. After 48 h, extracellular acidification rate (ECAR) **(A)** and oxygen consumption rate (OCR) **(B)** were measured using a Seahorse analyzer. During ECAR measurement, cells were sequentially treated with glucose, oligomycin, and 2-DG. During OCR measurements, cells were sequentially treated with oligomycin, fluorocarbonyl cyanide phenylhydrazone (FCCP), rotenone, and antimycin **(A)** Error bars indicate s.e.m (n = 6 wells per group). *p < 0.05, (unpaired two-tailed Student’s t-test). Data represent two independent experiments.

JunB-interacting transcription factors, BATF and IRF4, regulate metabolic reprogramming of glycolysis, which is closely associated with proliferation and differentiation of effector T cells (11, 37, 38). To address whether JunB is also involved in regulation of cellular metabolism, we activated *Junb*-deficient and control CD4^+^ T cells under differentiation conditions for Th1, Th2, and Th17 cells and measured extracellular acidification rate (ECAR) and oxygen consumption rate (OCR), which are indicative of glycolysis and oxidative phosphorylation, respectively, using a Seahorse analyzer. Loss of JunB did not cause obvious abnormalities in ECAR and OCR under Th1- and Th17-polarizing conditions (**Figures 5A, B**). In contrast, under Th2-polarizing conditions, loss of JunB slightly decreased basal ECAR, although it did not affect maximum glycolytic capacity (ECAR in the presence of oligomycin) and OCR (**Figures 5A, B**). These results suggest that JunB is largely dispensable for metabolic reprogramming of differentiating T helper cells, although it makes a minor contribution to glycolysis in Th2 cells.

### JunB-Dependent Transcriptional Control in T Helper Differentiation

**Figure 6:**
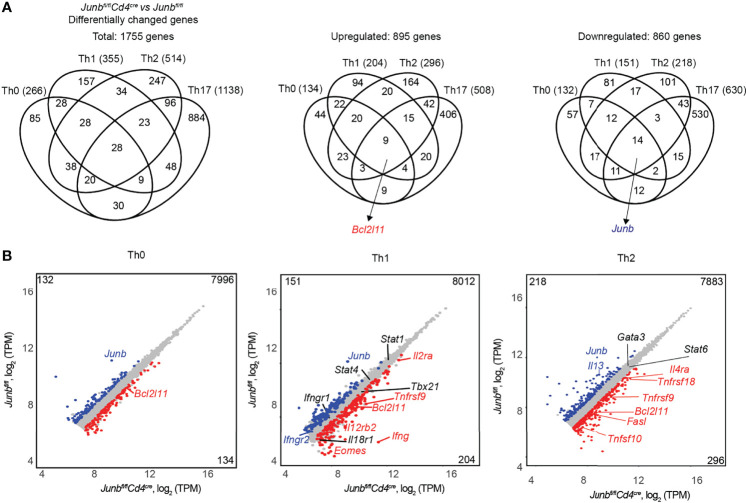
JunB-dependent transcriptional regulation in T helper cell differentiation. Naive CD4^+^ T cells isolated from *Junb^fl/fl^* or *Junb^fl/fl^Cd4^Cre^* mice were cultured under Th0-, Th1- and Th2-polarizing conditions for 48 h and subjected to RNA-seq analysis. Differentially expressed genes in *Junb*-deficient CD4^+^ T cells vs control cells (log_2_ fold change > 0.5 or < -0.5, *p* value < 0.05, base mean > 100 TPM (normalized transcript per kilobase million)) were identified in each Th-polarizing condition. **(A)** Venn diagrams show the overlap of differentially expressed genes identified in *Junb*-deficient CD4^+^ T cells vs control cells under different Th-polarizing conditions. RNA-seq data for *Junb*-deficient CD4^+^ T cells and control cells cultured under Th17-polarizing conditions were from GSE 98414. **(B)** Scatter plots represent genes differentially expressed in *Junb*-deficient CD4^+^ T cells vs control cells under differentiation conditions for Th0, Th1, and Th2 cells. Genes considered significantly upregulated or downregulated are highlighted in red and blue, respectively. Genes with insignificant changes are highlighted in grey. On each plot, left top and right bottom numbers indicate numbers of upregulated or downregulated genes, respectively.

To gain further insight into the role of JunB in differentiation of various T helper cells, we performed RNA-seq analysis of CD4^+^ T cells activated for 48 h under Th0-, Th1-, and Th2-poralizing conditions. Expressions of 266 genes in Th0 cells, 355 genes in Th1 cells, and 514 genes in Th2 cells were significantly affected by loss of JunB (**Figure 6A**). Combined with 1,138 differentially expressed genes identified in the previously reported RNA-seq data of *Junb*-deficient Th17 cells (20), we identified 1,755 genes that were upregulated or downregulated by the loss of JunB in T helper cell differentiation (**Figure 6A**). Most of those genes were found in a specific Th subset (85 genes for Th0 cells, 157 genes for Th1 cells, 247 genes for Th2 cells, 884 genes for Th17), while only 28 genes were found in all T helper cells tested (**Figure 6A**). Notably, *Bcl2l11* (encoding Bim) was one of 9 genes upregulated by loss of JunB under all Th-polarizing conditions tested (**Figure 6B**).

Gene ontology analysis of differentially expressed genes in *Junb*-deficient cells revealed that *immune system process*, *transcription process*, and *apoptotic process* were significantly affected by loss of JunB (**Supplementary Figure 4**). This analysis identified some apoptosis-related genes, including *Bcl2l11*, *Tnfsf9* (encoding 4-1-BBL), and *Tnfsf10* (encoding TRAIL), that were upregulated by JunB deletion in cells polarized under various conditions (**Figure 6B** and **Supplementary Figure 4**).

It has been shown that JunB inhibits IFN-γ expression in Th1 cells and promotes IL-4 expression in Th2 cells (20, 24), but contradictory results have also been reported (18). In our *in vivo* analysis, we observed an inhibitory function of JunB on IFN-γ expression, but IL-4 expression was not affected by loss of JunB (**Figures 2F, G**). On the other hand, our RNA-seq analysis showed that loss of JunB upregulated *Ifng*, *Il12rb2*, *Il2ra*, and *Eomes* in Th1 cells and *Il4ra* in Th2 cells, and downregulated *Il13* in Th2 cells (**Figure 6B**). Consistently, flow cytometry analysis showed that expression of IFN-γ and T-bet was significantly upregulated in *Junb*-deficient Th1 cells (**Supplementary Figures 5A, B**), while IL-4 and IL-13 were downregulated without affecting GATA3 expression in *Junb*-deficient Th2 cells (**Supplementary Figures 5A, B**). These data indicate that JunB inhibits expression of various Th1 signature molecules and promotes Th2 cytokines. Taken together, in addition to its role in regulation of lineage-specific molecule expression, JunB controls a subset of genes involved in apoptosis in differentiating T helper cells.

### JunB Directly Regulates Bim Expression by Interacting With IRF4

We and others have shown that JunB colocalizes with BATF and IRF4 at various gene loci containing AICE motif and thereby directly regulates expression of genes important for Th17 and effector Treg (eTreg) cells (14, 19–21, 39, 40). To extend our understanding of JunB function in diverse T helper cells, we performed ChIP-seq analysis for JunB, BATF, and IRF4 in Th1 cells. First, we analyzed whether JunB ChIP-seq peaks and AP-1-binding motifs were detected in genomic regions containing genes that were differentially expressed between *Junb*-deficient and control Th1 cells in RNA-seq data. We detected JunB ChIP-seq peaks or AP-1-binding motifs within ±100 kbp of transcription start sites (TSS) of the many differentially expressed genes, regardless of whether they were upregulated or downregulated by deletion of JunB (**Supplementary Figure 6A**), suggesting that JunB directly regulates transcription of those genes. Consistently, analysis using binding and expression target (BETA) software inferred that JunB had a directly regulatory potential in 37% of differentially expressed genes (**Supplementary Figure 6A**) (33). JunB’s direct target genes included various genes involved in differentiation and function of helper T subsets, including cytokines, chemokines, their receptors, and *Bcl2l11*. We next examined whether JunB ChIP-seq peaks overlapped with BATF and IRF4 ChIP-seq peaks in genomic regions containing direct JunB target genes (within ±100 kbp of the TSS). Overlapping ChIP-seq peaks for JunB, BATF, and IRF4 were detected in genomic regions for 72.8% of JunB direct target genes (**Supplementary Figure 6B**).

**Figure 7:**
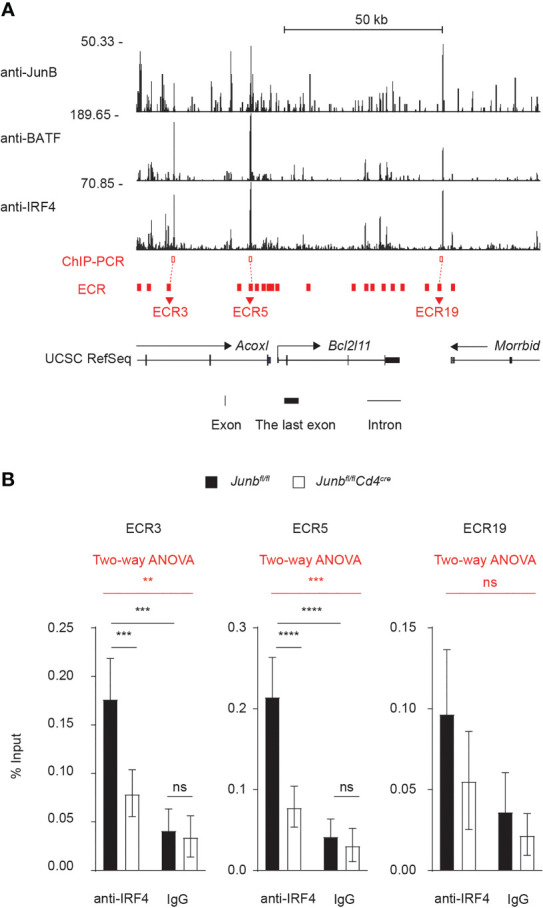
JunB is critical for IRF4 binding to the *Bcl2l11* locus **(A)** Naive CD4^+^ T cells from *Junb^fl/fl^* mice were activated under Th1-polarizing conditions for 48 h and subjected to ChIP-seq analysis using JunB, BATF, and IRF4 antibodies. ChIP-seq peaks for JunB, BATF, and IRF4 on *Bcl2l11* locus are shown. Three JunB/BATF/IRF4-colocalizing ChIP-seq peaks (red open box) were detected at regions close to evolutionarily conserved regions: ECR3, ECR5, and ECR19 of *Bcl2l11* locus (red filled box). Transcription directions (arrows), transcription start sites (TSS, turn right arrows), exons (black vertical lines and boxes) and introns (black solid lines) are indicated. **(B)** Naive CD4^+^ T cells from control (*Junb^fl/fl^*) or *Junb*-deficient (*Junb^fl/f^Cd4^Cre^*) mice were activated under Th1-polarizing conditions for 48 h and subjected to ChIP analysis using IRF4 antibodies. Eluted DNA was analyzed by qPCR using primers to detect genomic regions near ECR3, ECR5 and ECR19 (red open boxes shown in **(A)**. Error bars indicate s.e.m (n = 6 wells per group). The significance of IRF4 binding on each genomic region was first tested by using two-way ANOVA, as shown on the top of each graph and highlighted in red. The difference lying on each group was tested by using Tukey test, as shown within each graph. **p < 0.01, ***p < 0.001, ****p < 0.0001, ns non-significant (*p* > 0.05). Data represent a combination of two independent experiments.

Finally, we sought to understand molecular mechanisms underlying JunB-dependent control of Bim expression. We first identified 20 evolutionarily conserved regions (ECRs) located within ±50 kbp of *Bcl2l11* TSS (**Figure 7A**) by comparing human and mouse *Bcl2l11* loci with the evolutionarily conserved region browser (35). Three overlapping ChIP-seq peaks for JunB, BATF and IRF4 were found at the genomic regions proximal to ECR3, ECR5, and ECR19 (**Figure 7A**). We then evaluated whether JunB is required for DNA binding of IRF4 at those genomic regions of the *Bcl2l11* locus by ChIP-PCR analysis. Loss of JunB considerably diminished IRF4 binding to regions close to ECR3 and ECR5, but not ECR19, of the *Bcl2l11* locus (**Figure 7B**). In summary, JunB is required for IRF4-dependent inhibition of expression of pro-apoptotic molecules, including Bim. This inhibition is necessary for protection of clonally expanding T helper cells from apoptosis.

## Discussion

In this study, we demonstrate that JunB promotes clonal expansion of Th1, Th2 and Th17 cells both *in vitro* and *in vivo*. Clonal expansion and differentiation of various T helper cells depend on transcription factors IRF4 and BATF, but the role of JunB, a heterodimeric partner for BATF, in these processes had not been fully determined. Our data showed that accumulation of antigen-primed CD4^+^ T cells was significantly impaired by deletion of JunB in mice immunized with LPS, papain, or CFA, which predominantly induced Th1, Th2, and Th17 responses, respectively. Commensurate with this, viability of TCR-stimulated naïve CD4^+^ T cells was decreased by deletion of JunB under *in vitro* differentiation conditions for Th1, Th2, and Th17 cells. *Junb*-deficient CD4^+^ T cells were more sensitive to TCR-induced apoptosis with a concomitant increase of expression of a pro-apoptotic molecule, Bim, but their proliferation and metabolic reprogramming were comparable to controls. Bim was one of a few molecules whose expression was commonly upregulated by loss of JunB under Th1, Th2, and Th17-polarizing conditions and could plausibly be a mediator of apoptosis in *Junb*-deficient CD4^+^ T cells. Mechanistically, JunB promoted DNA-binding of IRF4, which is a negative regulator for Bim expression (8) at *Bcl2l11* CNS. Taken together, our data suggest that JunB is commonly required for survival of various T helper cells during their clonal expansion, plausibly by suppressing expression of pro-apoptotic Bim.

Our *in vitro* data showed that degrees of JunB-dependent suppression of apoptosis were dependent on the cytokine environment that facilitates various T helper cells. Under Th0-polarizing conditions, there was no increase in apoptosis despite significant upregulation of Bim expression. In addition, the degree of increase in apoptosis induced by JunB deficiency under Th1-polarizing conditions was less prominent than under Th2- and Th17-polarizing conditions. On the other hand, there was no significant difference in Bim/Bcl2 ratios among different Th subsets deficient for JunB. Taken together, these data suggest that changes in not only the Bim/Bcl2 ratio but also the expression and/or activity of other apoptosis regulators may cause the increase of apoptosis in *Junb*-deficient T helper cells. Indeed, in our RNA-seq analysis, we found that several apoptosis regulators were differentially expressed in a manner dependent on Th-polarizing conditions. In the future, functional analysis of these apoptosis regulators should provide further mechanistic insights into how JunB regulates apoptosis in differentiating T helper cells.

JunB also regulates expression of a variety of lineage-specific genes in a context-dependent manner. As we and others have reported, JunB promotes expression of *Rorc*, *Il17a* and *Il17f* (19, 20) in Th17 cells and negatively regulates *Ifng* in Th1 and Th17 cells (20). In this study, our RNA-seq analysis further clarified the role of JunB in a transcriptional program for T helper differentiation. Expression of Th1-related genes, not only *Ifng*, but also *Il12rb2*, *Il2ra* and *Eomes*, was promoted by JunB deletion, while expression of Th2-related genes, *Il4ra* and *Il13*, was upregulated and downregulated, respectively. This observation is consistent with previous studies that reported an increase of IFN-γ and decreases of IL-4 and IL-13 in *Junb*-deficient Th1 and Th2 cells (20, 24). Although defective clonal expansion of *Junb*-deficient CD4^+^ T cells makes data interpretation difficult, our *in vivo* data showed that the percentage of LPS-induced, IFN-γ-expressing cells increased, while percentages of papain-induced IL-4 and IL-13 expressing cells were unaltered. Inconsistency between *in vitro* and *in vivo* results of Th2 cytokine expression in *Junb*-deficient cells may be due to a context-dependent requirement for JunB in Th2 differentiation, which is reminiscent of our previous observation that JunB is required for pathogenic Th17 differentiation, but not homeostatic Th17 differentiation (19).

Since loss of *Junb* also greatly sensitizes thymus-derived Treg cells to TCR-induced apoptosis (21), CD4^+^ helper and regulatory T cells share a common requirement for JunB-dependent negative modulation of the TCR-induced apoptosis signal. However, in some situations, T helper cells can be generated normally, independently of JunB. For example, loss of JunB does not affect the frequency of gut-resident homeostatic Th17 cells (19, 20). We still do not know what determines the necessity of JunB in Th cell generation, but JunB may not be needed for accumulation of cells that are activated by a weak TCR signal or in a less inflammatory environment.

In conclusion, we provide substantial evidence that JunB is critical, not only in Th17, but also in Th1 and Th2 responses both *in vitro* and *in vivo*. Our data shed light on a transcriptional regulatory mechanism that is commonly required for clonal expansion of various CD4^+^ T helper cells.

## Data Availability Statement

The datasets presented in this study can be found in online repositories. The names of the repository/repositories and accession number(s) can be found below: Gene Expression Omnibus, accession ID: GSE172490.

## Ethics Statement

The animal study was reviewed and approved by OIST Animal Experiment Review Committee.

## Author Contributions

TH designed, performed, and analyzed the majority of experiments, performed computation analyses, and wrote the manuscript. DS, NT, and SS performed and supported experiments. HC supported computational analyses. MM and YS maintained mice and performed genotyping. HI wrote and edited the manuscript. All authors contributed to the article and approved the submitted version.

## Funding

This work was supported by KAKENHI grant (18K15200, 19K22547) and by OIST Graduate University.

## Conflict of Interest

We declare that the research was conducted in the absence of any commercial or financial relationships that could be construed as a potential conflict of interest.

## Publisher’s Note

All claims expressed in this article are solely those of the authors and do not necessarily represent those of their affiliated organizations, or those of the publisher, the editors and the reviewers. Any product that may be evaluated in this article, or claim that may be made by its manufacturer, is not guaranteed or endorsed by the publisher.
