# Supplementary material for: JunB Is Critical for Survival of T Helper Cells
Source: Front Immunol. 2022 Jun 28;13:901030. doi: 10.3389/fimmu.2022.901030 (PMC9273772; doi:10.3389/fimmu.2022.901030)
Supplement: Supplementary file 1 [file DataSheet_1.pdf]

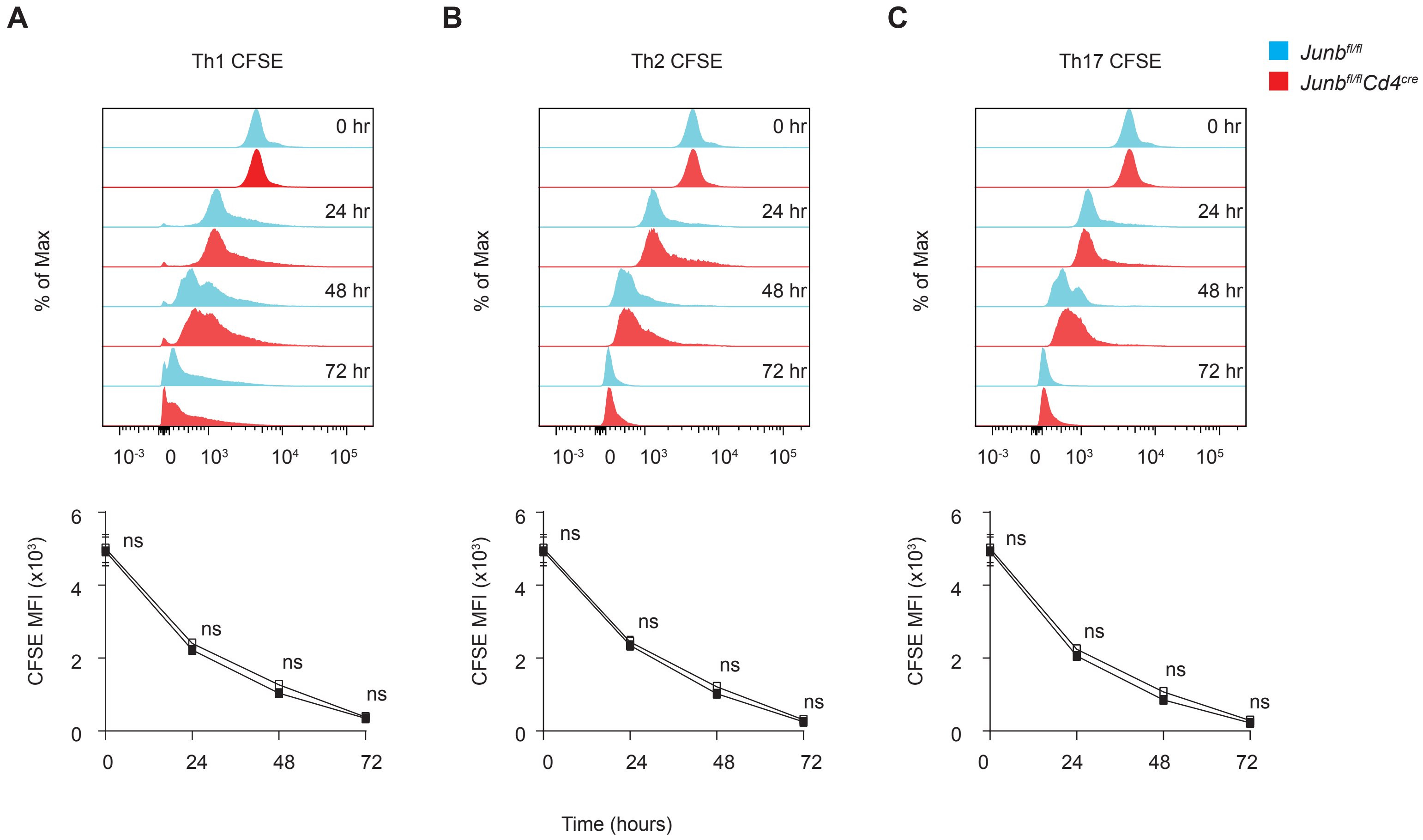

Supplementary Fig. 1. JunB is not essential for proliferation of differentiating T helper cells *in vitro*. Naive CD4$^+$ T cells isolated from *Junb*$^{fl/fl}$ or *Junb*$^{fl/fl}$*Cd4*^{cre} mice were cultured under the indicated polarizing conditions for 72 h. Flow cytometry analysis of expression of CFSE MFI at indicated time points for Th1 (**A**) , Th2 (**B**) , and Th17 (**C**) cells. (A-C) Error bars indicate s.d (n = 3-4 wells per group). ns non-significant, (unpaired two-tailed Student's t-test). Data represents two independent experiments.

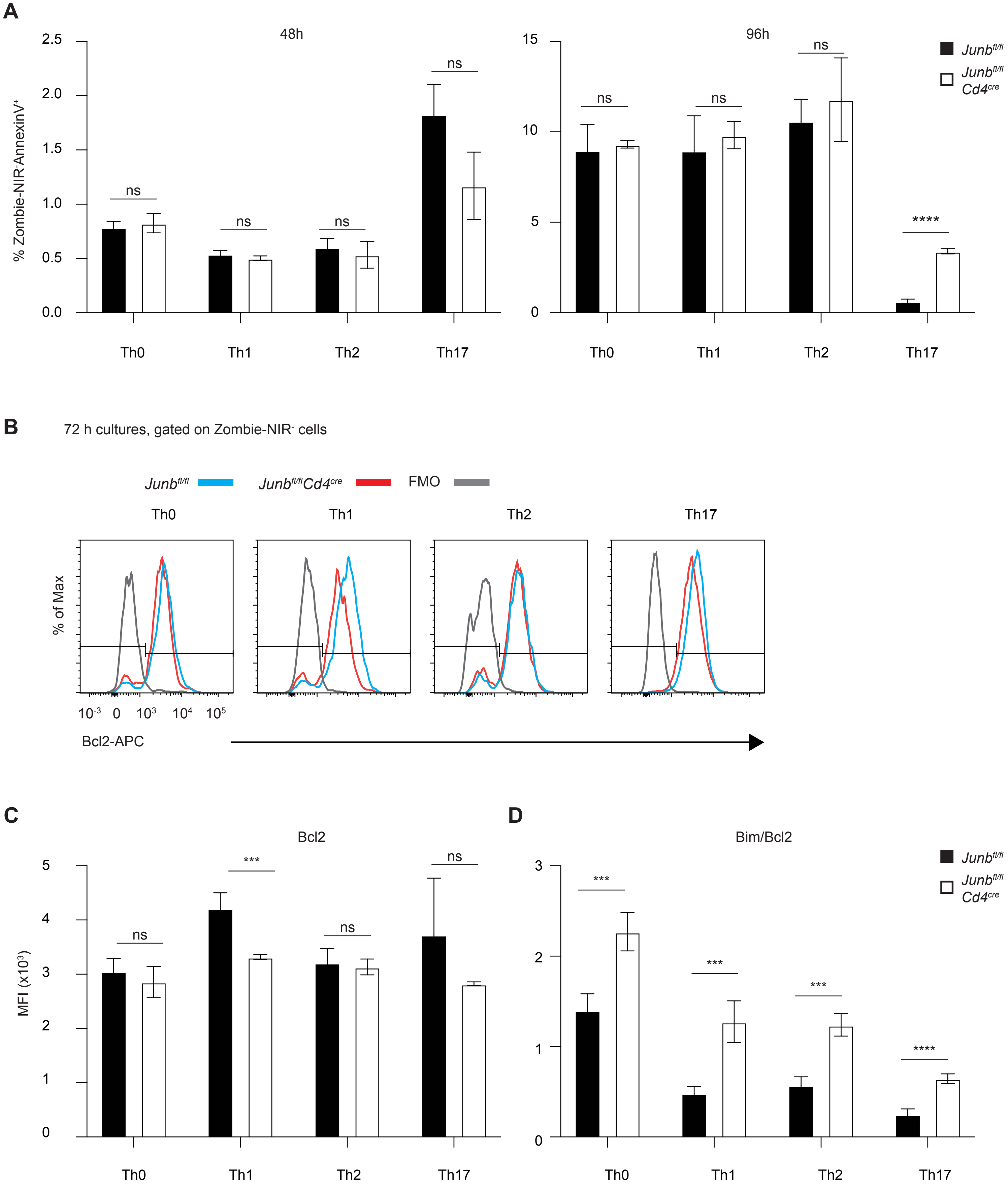

Supplementary Fig. 2. Induction of apoptosis in various T helper subsets.
(**A-D**) Naive CD4$^+$ T cells isolated from *Junb$^{fl/fl}$* or *Junb$^{fl/fl}$Cd4^{cre}* mice were activated under Th0-, Th1-, Th2- and Th17-polarizing conditions. (**A**) Flow cytometry analysis of Zombie-NIR and Annexin-V staining of cells at indicated time points. (B) Flow cytometry analysis of expression of Bcl2 at 72 h after cell activation. (**C**) The bar graph shows the mean fluorescence intensity (MFI) of Bcl2 expression in Zombie-NIR$^-$ cells. (**D**) The ratio of expression (MFI) of Bim to Bcl2. The expression of Bim was analyzed in Fig. 4C. (A-D) Error bars indicate s.d. (n = 3 wells per group). * $p < 0.05$, ** $p < 0.01$, *** $p < 0.001$, **** $p < 0.0001$, (unpaired two-tailed Student's t-test).

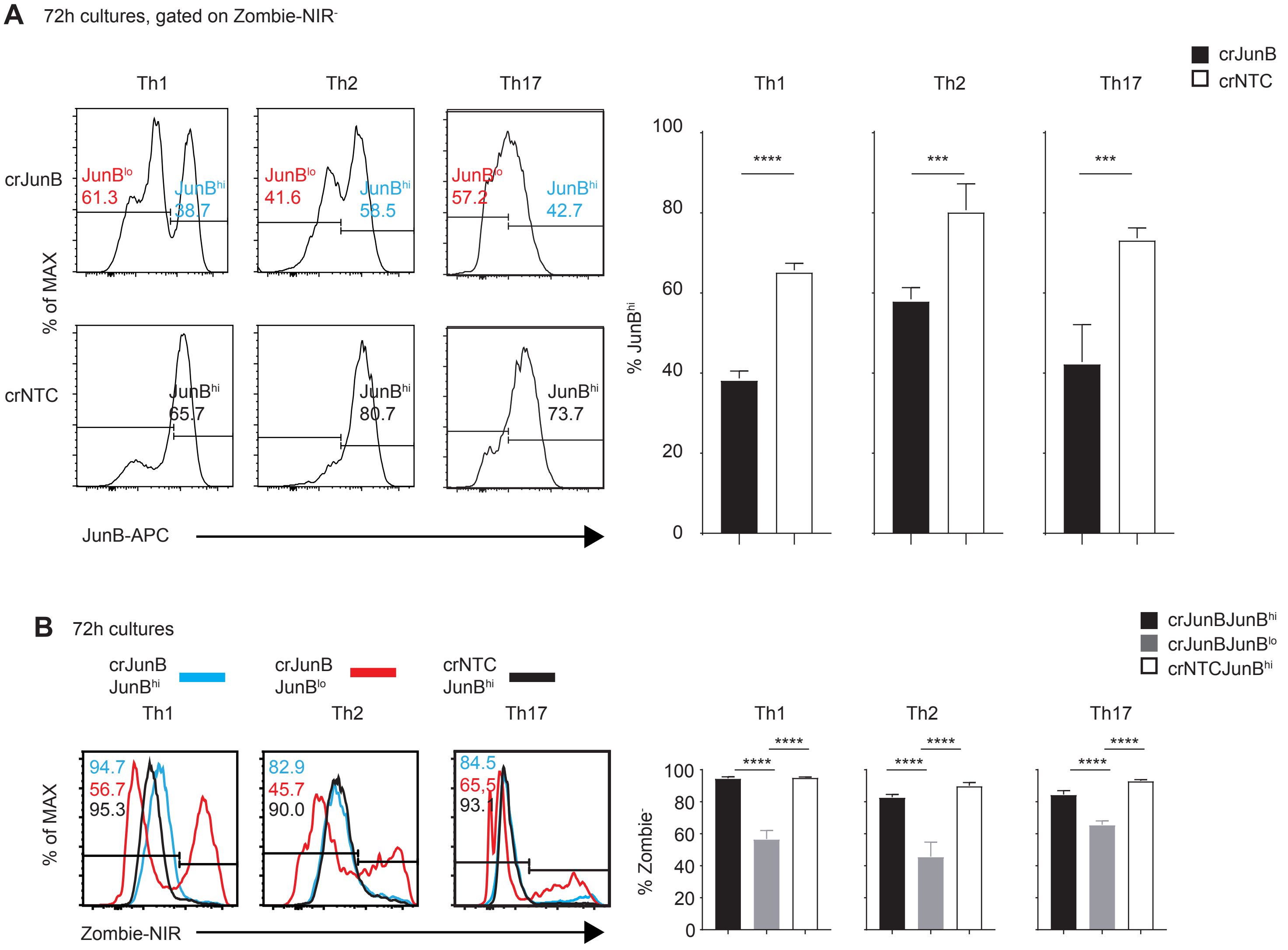

Supplementary Fig. 3. CRISPR-mediated JunB knockout impaired survival of TCR-stimulated CD4$^+$ T cells. Naive CD4$^+$ T cells isolated from C57BL/6 mice were electroporated with Cas9 protein together with Alt-R crRNA targeting *Junb* (crJunB) or negative control Alt-R crRNA (crNTC). After nucleofection, cells were rested in IMDM containing IL-7 for 72 h and then activated under Th1-, Th2- and Th17-polarizing conditions for another 72 hours. (**A**) Flow cytometry analysis of JunB. Numbers in contour plots indicate average percentages of cells exhibiting high or low JunB expression levels (JunB^{hi}, JunB^{lo}). (**B**) Flow cytometry analysis of Zombie-NIR. Error bars indicate s.d. (n = 4-6 wells per group). * $p < 0.05$, ** $p < 0.01$, *** $p < 0.001$, **** $p < 0.0001$, (unpaired two-tailed Student's t-test).

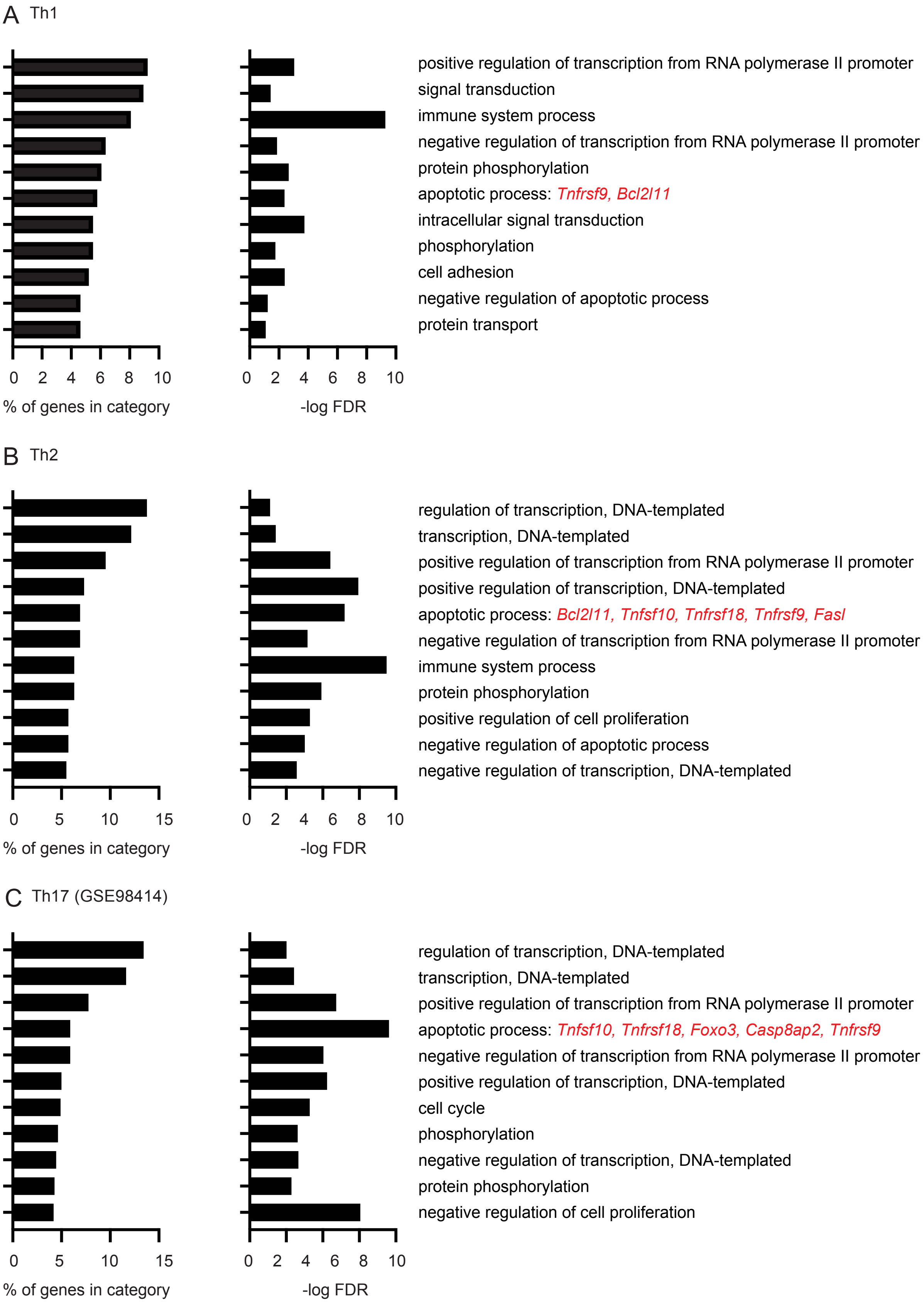

Supplementary Fig. 4. DAVID GO (gene ontology) pathway analysis for JunB target genes. Naïve CD4^{+} T cells isolated from *Junb*$^{fl/fl}$ or *Junb*$^{fl/fl}$*Cd4*^{cre} mice were cultured under Th1- and Th2-polarizing conditions for 48 h and subjected to RNA-seq analysis. Differentially expressed genes in *Junb*-deficient CD4^{+} T cells vs control cells (log2 fold change > 0.5, p value < 0.05, base mean > 100 TPM (normalized transcript per kilobase million)) were identified in each Th-polarizing condition. (A-C) DAVID GO biological process analysis of genes differentially expressed in *Junb*-deficient CD4^{+} T cells vs control cells under differentiation conditions for Th1 (**A**) and Th2 (**B**). RNA-seq data for *Junb*-deficient CD4^{+} T cells cultured under Th17-polarizing conditions were from GSE 98414 (**C**). Representative genes for apoptotic process are shown.

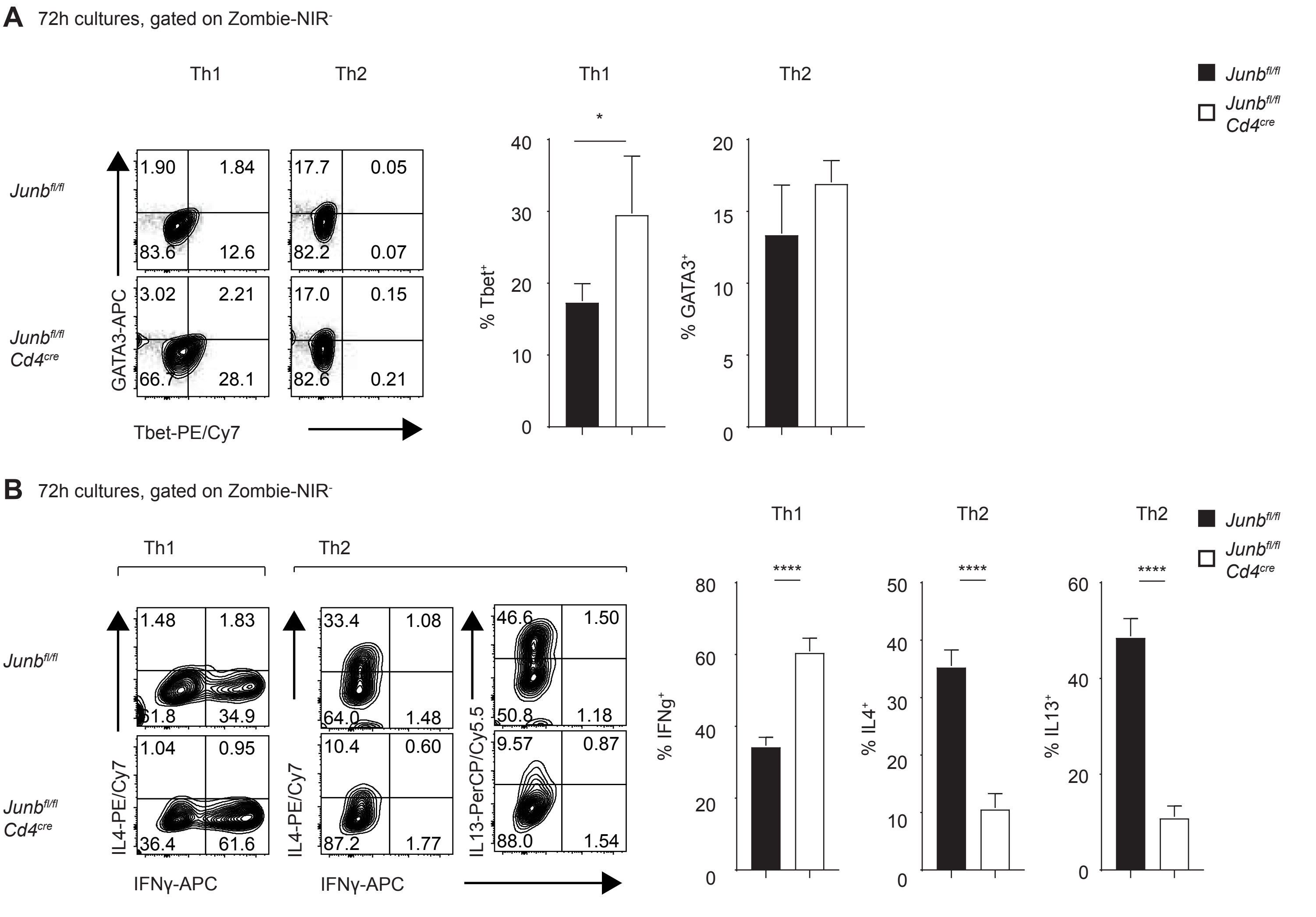

Supplementary Fig. 5. JunB regulates expression of the lineage-specific genes in Th1 and Th2 cells. Naive CD4+ T cells isolated from*Junb*$^{fl/fl}$ or *Junb*$^{fl/fl}$*Cd4*^{cre} mice were cultured under Th1- and Th2-polarizing conditions for 72 h. Flow cytometry analysis of expression of lineage-specifying transription factors (A) and cytokines (B) for Th1 and Th2 cells. Error bars indicate s.d. (n = 4-6 wells per group). * $p < 0.05$, ** $p < 0.01$, *** $p < 0.001$, **** $p < 0.0001$, (unpaired two-tailed Student's t-test).

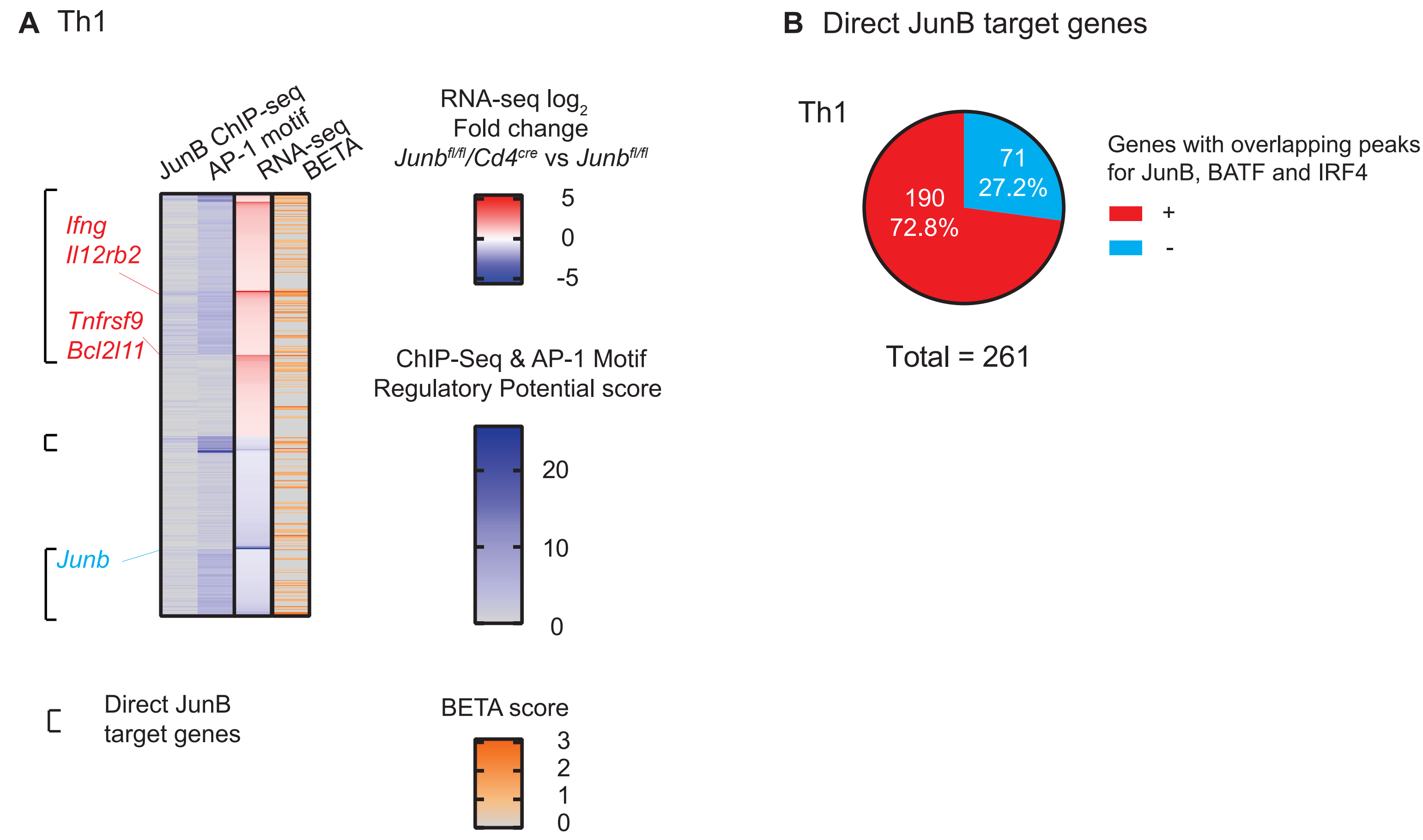

Supplementary Fig. 6. Prediction of direct JunB target genes.
ChIP-seq analysis of JunB, AP-1 motif analysis, RNA-seq analysis of genes sigficantly differentially expressed in *Junb*-deficient ($Junb^{fl/fl}Cd4^{cre}$) $CD4^+$ T cells vs control cells ($Junb^{fl/fl}$) (JunB target genes), and BETA analysis in Th1 cells, as shown in the heatmap. The order of genes in the heatmap was determined by unsupervised k-means clustering (K = 4). The JunB ChIP-seq peak dataset was obtained from JunB ChIP-seq data for Th1 cells. The AP-1 motif dataset was obtained by scanning AP-1 binding motifs across the mouse genome (UCSC mm10). RNA-seq anaysis and the criteria of JunB target genes was as described in Supplementary Fig. 5. JunB target genes that had JunB ChIP-seq peaks and/or AP-1-binding motifs within 100 kb of their TSS were defined as “direct JunB target genes.” BETA software was used to confirm the direct JunB target genes. Genes with non-zero BETA scores have a great likelihood of direct regulation by JunB. (B) Pie charts show the percentage of JunB direct target genes that did or did not have overlapping peaks for JunB, BATF, and IRF4. Overlapping peaks were obtained by comparing ChIP-seq data of JunB with that of BATF and IRF4. BATF and IRF4 ChIP-seq peak datasets were obtained from the ChIP-seq data for Th1 cells.

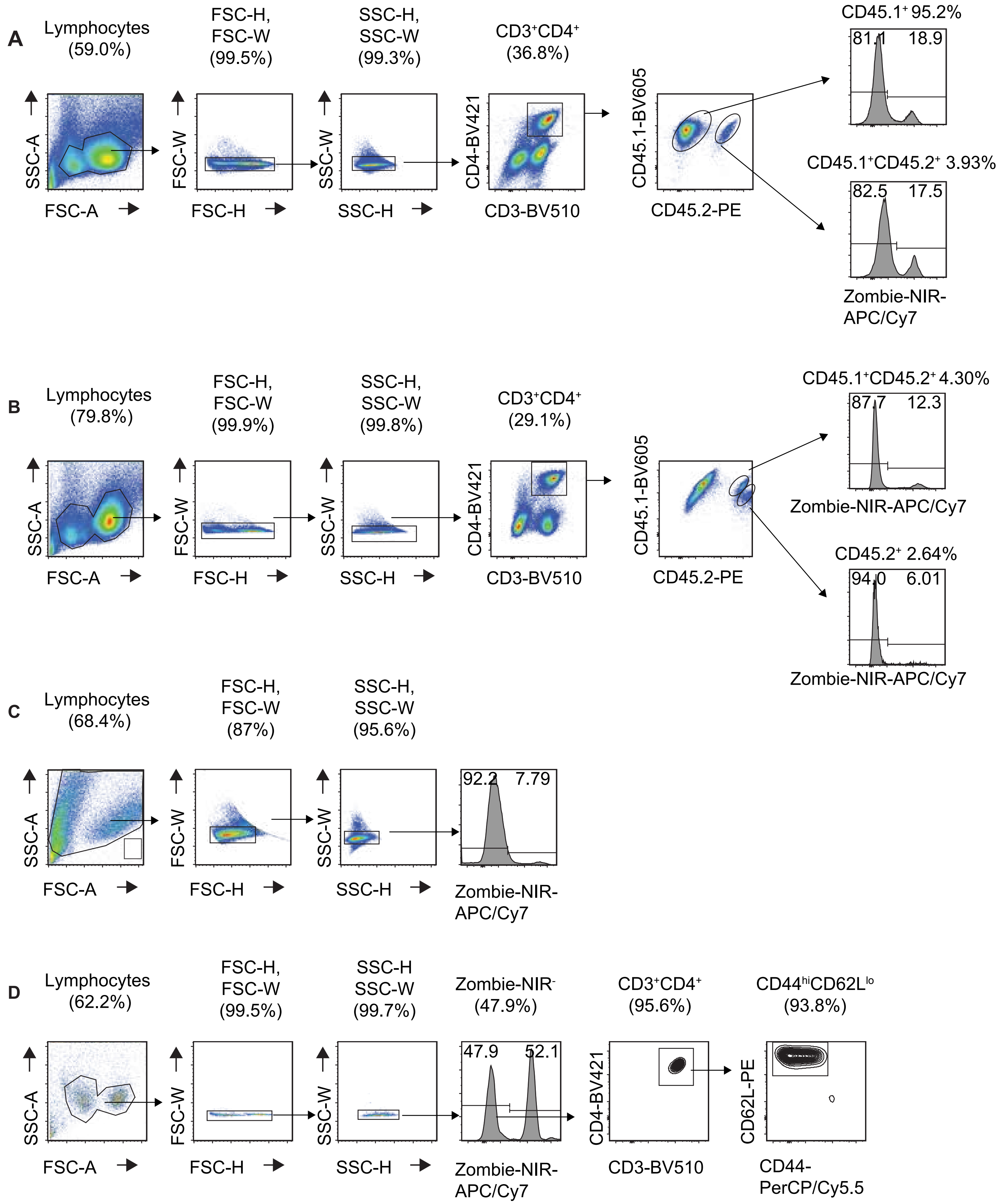

Supplementary Fig. 7. Gating strategies for flow cytometry analysis.
(**A**) Gating strategies for flow cytometry analysis of transferred OT-II cells (CD45.1^{+}45.2^{+}) and recipient CD4^{+} T cells (CD45.1^{+}), related to Fig. 1. (**B**) Gating strategies for flow cytometry analysis of OT-II cells (CD45.1^{+}45.2^{+}) and co-transferred congenic OT-II cells (CD45.1^{+}45.2^{+}), related to Fig. 2 and Fig. 3. (**C**) Gating strategies for all *in vitro* polarizing cultures, related to Fig. 4, Supplementary Fig. 1, Supplementary Fig. 2, and Supplementary Fig. 5. (**D**) Flow cytometry analysis of the purity of murine naïve CD4^{+} T (CD4^{+}CD25^{-}CD62L^{hi}CD44^{lo}) cells.
